# Radiotherapy Patterns of Care for Locally-advanced Non-small Cell Lung Cancer in the Pre- and Post-durvalumab Era: A Region-wide Survey in a Japanese Prefecture

**DOI:** 10.1093/jrr/rrab116

**Published:** 2021-12-30

**Authors:** Nobuteru Kubo, Daijiro Kobayashi, Mototaro Iwanaga, Masana Matsuura, Keiko Higuchi, Jun Eishima, Hiroyuki Muramatsu, Naoko Okano, Mariko Shioya, Masahiro Onishi, Tetsuya Aoki, Takahiro Oike, Tatsuya Ohno, Gunma Society for Radiation Oncology (GUSTRO)

**Affiliations:** Department of Radiation Oncology, Gunma University Graduate School of Medicine, 3-39-22, Showa-machi, Maebashi, Gunma 371-8511, Japan; Gunma University Heavy Ion Medical Center, 3-39-22, Showa-machi, Maebashi, Gunma 371-8511, Japan; Department of Radiation Oncology, Gunma Prefectural Cancer Center, 617-1, Takahayashi-nishicho, Ota, Gunma 373-8550, Japan; Department of Radiation Oncology, Japanese Red Cross Maebashi Hospital, 389-1 Asakura-machi, Maebashi, Gunma 371-0811, Japan; Department of Radiation Therapy, National Hospital Organization Shibukawa Medical Center, 383 Shirai, Shibukawa, Gunma 377-0280, Japan; Department of Radiation Oncology, Isesaki Municipal Hospital, 12-1, Tsunatorimoto-machi, Isesaki, Gunma, 372-0817, Japan; Department of Radiation Oncology, National Hospital Organization Takasaki General Medical Center, 36, Takamatsu-cho, Takasaki, Gunma 370-0829, Japan; Department of Radiology, Kiryu Kosei General Hospital, 6-3, Orihime-cho, Kiryu, Gunma 376-0024, Japan; Department of Radiation Oncology, Gunma University Graduate School of Medicine, 3-39-22, Showa-machi, Maebashi, Gunma 371-8511, Japan; Department of Radiotherapy, Public Tomioka General Hospital, 2073-1, Tomioka, Tomioka, Gunma, Japan; Department of Radiation Oncology, Fujioka General Hospital, 813-1, Nakakurisu, Fujioka, Gunma 375-8503, Japan; Oncology Center, Hidaka Hospital, 886, Nakao-machi, Takasaki, Gunma 370-0001, Japan; Department of Radiation Oncology, Tatebayashi Kosei General Hospital, 262-1 Narushima-cho, Tatebayashi, Gunma 374-8533, Japan; Department of Radiation Oncology, Gunma University Graduate School of Medicine, 3-39-22, Showa-machi, Maebashi, Gunma 371-8511, Japan; Department of Radiation Oncology, Gunma University Graduate School of Medicine, 3-39-22, Showa-machi, Maebashi, Gunma 371-8511, Japan; Gunma University Heavy Ion Medical Center, 3-39-22, Showa-machi, Maebashi, Gunma 371-8511, Japan

**Keywords:** non-small cell lung carcinoma (NSCLC), concurrent chemoradiotherapy (CCRT), durvalumab, patterns of care

## Abstract

The promising results of the PACIFIC study led to the approval of consolidation durvalumab for coverage by the National Health Insurance (NHI) in 2018 for patients with locally-advanced unresectable non-small cell lung carcinoma (NSCLC) treated with definitive concurrent chemoradiotherapy (CCRT). However, the effect of NHI coverage on the patterns of care for this population remains unclear. Here, we conducted a questionnaire-based survey to determine the patterns of care for patients with stage II–III NSCLC treated with definitive radiotherapy in 2017 (pre-durvalumab era) or in 2019 (post-durvalumab era). Data were obtained from 11 radiotherapy facilities in Gunma prefecture, which has a population of 1.94 million. We identified 80 and 83 patients with stage II–III NSCLC who received definitive radiotherapy in Gunma in 2017 and 2019, respectively. At a given facility, CCRT was the treatment of choice in a significantly greater proportion of patients in 2019 than in 2017 (66% ± 20% vs 51% ± 29%, *P* = 0.041). Intensity-modulated radiotherapy (IMRT) was more frequent in 2019 than in 2017 (24% vs 1.2%). Carboplatin plus paclitaxel was used for CCRT at higher rate in 2019 than in 2017 (73% vs 44%). Consolidation durvalumab was performed in 73% (40/55) of CCRT-treated patients in 2019, and the treatment was performed for the planned 12 months in 45% (18/40) of patients. These data indicate that NHI coverage of durvalumab might be a possible reason for choosing CCRT in patients with stage II–III NSCLC in the real-world setting.

## INTRODUCTION

Lung cancer is the leading cause of death among all cancers [1]. Non-small cell lung cancer (NSCLC) accounts for approximately 85% of all lung cancers [2]. In 2017, the randomized phase 3 PACIFIC trial showed that consolidation therapy with durvalumab, an anti-programmed death ligand 1 antibody, significantly prolongs progression-free survival in patients with stage III NSCLC treated with platinum-based chemoradiotherapy [3]. Based on this study, concurrent chemoradiotherapy (CCRT) followed by durvalumab is recommended as the standard definitive treatment for locally-advanced unresectable NSCLC [4]. In 2018, durvalumab was approved for coverage by the National Health Insurance (NHI) of Japan for patients with locally-advanced unresectable NSCLC treated with definitive CCRT, which enabled patients to receive durvalumab treatment without a significant financial burden.

In the clinical setting, it is estimated that not a small number of patients with locally-advanced NSCLC has been treated with modalities other than CCRT (including radiotherapy alone and sequential chemoradiotherapy) because of medical reasons, including advanced age, poor performance status and renal dysfunction. These patients do not benefit from consolidation durvalumab. However, the patterns of care in patients with locally-advanced unresectable NSCLC remain unclear. The impact of the NHI coverage of durvalumab on the patterns of care in the real-world setting is also unclear. To address these issues, we conducted a region-wide questionnaire-based survey in a prefecture of 1.94 million people.

## MATERIALS AND METHODS

### Questionnaire-based survey

This study involved patients with stage II–III NSCLC treated with definitive radiotherapy in 2017 (pre-durvalumab era) or in 2019 (post-durvalumab era). An electronic questionnaire was used to collect data on the patterns of care in eligible patients treated in all the 11 radiotherapy facilities located in Gunma prefecture, Japan, on 31 March 2021.

The questionnaire consisted of the following items: (i) patient characteristics including age, performance status, gender, tumor-node-metastasis (TNM) classification and stage (based on 8th edition) [5], primary tumor site and tumor histology, (ii) radiotherapy data including date of initiation and completion, technique, total dose and fractionation, mean lung dose and V_5_ and V_20_ for the lung excluding gross tumor volume (lung minus GTV) (V_X_ indicates the volume that received at least X Gy), (iii) chemotherapy data including regimen, combination pattern with radiotherapy (i.e. CCRT or not) and the reason for not implementing CCRT, (iv) durvalumab data including date of initiation and completion, the number of sessions and the reason for not implementing durvalumab or that for discontinuance, and (v) adverse events including maximum grade for pneumonitis and date of onset for pneumonitis ≥Grade 2 (assessed based on the Common Terminology Criteria for Adverse Events version 4.0 [CTCAE]). It is practically impossible to distinguish pneumonitis induced by CCRT from that induced by durvalumab. Therefore, we collected data on treatment-associated pneumonitis without the distinction.

Collected data were analyzed by stratifying the patients into CCRT and non-CCRT groups. Because this stratification was based on the eligibility for durvalumab (i.e. history of definitive CCRT), the non-CCRT group included patients treated with radiotherapy alone and those treated with sequential chemoradiotherapy. Definitive radiotherapy was defined as radiotherapy at a total dose ≥50 Gy. Patients were followed-up for at least 1 year after the completion of radiotherapy.

### Statistical analysis

The association of patient characteristics with patient groups was examined by Fisher’s exact test. Intra-facility differences in the proportion of CCRT-treated patients between 2017 and 2019 was examined by the Wilcoxon signed rank test. Differences in dose volume parameters between two groups were examined by the Mann–Whitney U test. Differences in dose volume parameters between three or more groups were examined by the Kruskal–Wallis test. All statistical analyses were performed using GraphPad Prism 8 (GraphPad Software, San Diego, CA, USA). A *P* value <0.05 was considered statistically significant.

## RESULTS

We identified 80 and 83 patients with stage II–III NSCLC who were treated with definitive radiotherapy in Gunma prefecture in 2017 and 2019, respectively (Table 1). Patients treated with CCRT were significantly younger than non-CCRT patients in 2017 and 2019 (*P* < 0.001 for both years). There were no significant differences in performance status, gender, T factor, N factor, stage, histology and primary tumor site between the two groups. In 2017, the proportion of patients treated with concurrent chemotherapy was comparable to that of those without (41 and 39 patients, respectively). In 2019, two thirds (55/83) of the patients were treated with CCRT. CCRT was the treatment of choice for a significantly greater proportion of patients in 2019 than in 2017 (66 ± 20% vs 51 ± 29%, *P* = 0.041) (Table 2). These data indicate that the NHI coverage of durvalumab affected the choice of CCRT in patients with stage II–III NSCLC.

**Table 1:** Characteristics of patients with stage II–III NSCLCs treated with definitive radiotherapy in Gunma prefecture.

Characteristics	2017		2019	
	CCRT	non-CCRT	CCRT	non-CCRT
	(*n* = 41)	(*n* = 39)	(*n* = 55)	(*n* = 28)
Age	68 (45–86)	80 (53–90)	70 (38–83)	79 (63–91)
Performance status				
*0*	21 (51)	7 (18)	25 (45)	4 (14)
*1*	18 (44)	19 (49)	25 (45)	12 (43)
*2*	1 (2)	11 (28)	5 (9)	10 (36)
*3*	0 (0)	1 (3)	0 (0)	2 (7)
*4*	0 (0)	1 (3)	0 (0)	0 (0)
*Unknown*	1 (2)	0 (0)	0 (0)	0 (0)
Gender				
*Male*	26 (63)	30 (77)	46 (84)	19 (68)
*Female*	15 (37)	9 (23)	9 (16)	9 (32)
T				
*1*	7 (17)	2 (5)	12 (22)	1 (4)
*2*	7 (17)	11 (28)	9 (16)	9 (32)
*3*	13 (32)	14 (36)	16 (29)	9 (32)
*4*	14 (34)	12 (31)	16 (29)	8 (29)
*Unknown*	0 (0)	0 (0)	2 (4)	1 (4)
N				
*0*	4 (10)	12 (31)	4 (7)	10 (36)
*1*	3 (7)	7 (18)	4 (7)	3 (11)
*2*	30 (73)	17 (44)	31 (56)	11 (39)
*3*	4 (10)	3 (8)	16 (29)	4 (14)
Stage				
*IIA*	0 (0)	3 (8)	0 (0)	4 (14)
*IIB*	1 (2)	10 (26)	2 (4)	4 (14)
*IIIA*	18 (44)	11 (28)	22 (40)	9 (32)
*IIIB*	20 (49)	13 (33)	22 (40)	9 (32)
*IIIC*	2 (5)	2 (5)	9 (16)	2 (7)
Histology				
*Sq cell carcinoma*	18 (44)	18 (46)	15 (27)	16 (57)
*Adenocarcinoma*	20 (49)	11 (28)	27 (49)	5 (18)
*NSCLC, NOS*	1 (2)	1 (3)	9 (16)	1 (4)
Others^*^	2 (5)	2 (5)	4 (7)	0 (0)
*Unknown*	0 (0)	7 (18)	0 (0)	6 (21)
Tumor site				
*Right upper lobe*	12 (29)	11 (28)	19 (35)	8 (29)
*Right middle lobe*	3 (7)	2 (5)	2 (4)	2 (7)
*Right lower lobe*	9 (22)	11 (28)	11 (20)	6 (21)
*Left upper lobe*	13 (32)	10 (26)	15 (27)	9 (32)
*Left lower lobe*	3 (7)	1 (3)	5 (9)	1 (4)
*Hilar / mediastinal*	1 (2)	4 (10)	3 (5)	2 (7)

Data are shown as numbers (%) except age, for which the median (range) is shown. CCRT, concurrent chemoradiotherapy; NSCLC, non-small cell lung carcinoma; Sq, squamous; NSCLC, NOS, non-small cell lung carcinoma, not otherwise specified. * large cell neuroendocrine carcinoma (n = 3), poorly differentiated carcinoma (n = 2), adenosquamous carcinoma (n = 1), adenoid cystic carcinoma (n = 1), clear cell carcinoma (n = 1).

**Table 2:** Patterns of radiotherapy.

Facility	2017		2019	
	CCRT	non-CCRT	CCRT	non-CCRT
#1	15 (79)	4 (21)	10 (59)	7 (41)
#2	5 (33)	10 (67)	10 (91)	1 (9)
#3	7 (50)	7 (50)	4 (50)	4 (50)
#4	2 (40)	3 (60)	7 (78)	2 (22)
#5	2 (25)	6 (75)	2 (50)	2 (50)
#6	0 (0)	2 (100)	6 (60)	4 (40)
#7	3 (75)	1 (25)	3 (50)	3 (50)
#8	1 (25)	3 (75)	4 (80)	1 (20)
#9	5 (83)	1 (17)	2 (100)	0 (0)
#10	0 (0)	0 (0)	6 (75)	2 (25)
#11	1 (33)	2 (67)	1 (33)	2 (67)
Total	41 (51)	39 (49)	55 (66)	28 (34)

Number (%) is shown. NSCLC, non-small cell lung carcinoma; CCRT, concurrent chemoradiotherapy.

**Fig. 1:**
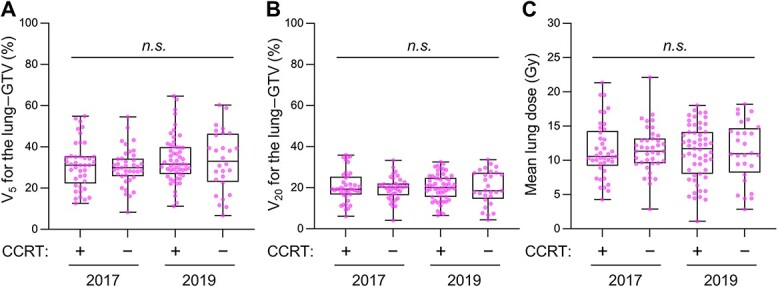
Comparison of dose volume parameters for the lung according to treatment modality and treatment year in patients with stage II–III NSCLC treated with definitive radiotherapy. **A.** V_5_ for the lung minus GTV. **B.** V_20_ for the lung minus GTV. **C.** Mean lung dose. CCRT, concurrent chemoradiotherapy. *n.s.* indicates no statistical significance assessed by Kruskal–Wallis test.

Regarding the radiotherapy technique, in 2017, almost all patients were treated using 3-dimensional conformal radiotherapy (3DCRT) regardless of the presence or absence of concurrent chemotherapy (100% and 97%, respectively) (Table 3). In 2019, by contrast, intensity-modulated radiotherapy (IMRT) was used alone or in combination with 3DCRT in approximately one in four patients regardless of the presence or absence of concurrent chemotherapy (26% and 22%, respectively) (Table 3). In both 2017 and 2019, most of the patients who underwent CCRT (90% and 96%, respectively) received a total dose of 60 Gy. There were no significant differences in V_5_ for the lung minus GTV, V_20_ for the lung minus GTV, and the mean lung dose (*P* = 0.50, 0.96 and 0.99, respectively) between the different treatment groups or between the treatment years (Fig. 1). There were no significant differences in the dose-volume parameters for the lung between 3DCRT and IMRT (data not shown).

**Table 3:** Details of radiotherapy.

RT details	2017		2019	
	CCRT	non-CCRT	CCRT	non-CCRT
	(*n* = 41)	(*n* = 39)	(*n* = 55)	(*n* = 28)
Technique				
*3DCRT*	41 (100)	38 (97)	40 (73)	22 (79)
*3DCRT + IMRT*	0 (0)	0 (0)	6 (11)	1 (4)
*IMRT*	0 (0)	1 (3)	8 (15)	5 (18)
*Unknown*	0 (0)	0 (0)	1 (2)	0 (0)
Total dose (Gy)				
*50–59*	1 (2)	1 (3)	2 (4)	0 (0)
*60*	37 (90)	16 (41)	53 (96)	10 (36)
*61–69*	3 (7)	15 (38)	0 (0)	16 (57)
*70+*	0 (0)	7 (18)	0 (0)	2 (7)

Number (%) is shown. NSCLC, non-small cell lung carcinoma; CCRT, concurrent chemoradiotherapy; RT, radiotherapy; 3DCRT, 3-dimensional conformal radiotherapy; IMRT, intensity-modulated radiotherapy.

Regarding concurrent chemotherapy, platinum-doublet regimens, which are the standard treatment [6, 7, 8, 9], were used predominantly in both 2017 and 2019, and carboplatin plus paclitaxel was used at higher rate in 2019 than in 2017 (73% vs 44%) (Supplementary Table 1). Daily carboplatin was the predominant treatment in elderly patients (data not shown) [10]. Advanced age was the most frequent reason for not implementing CCRT, followed by complications and poor performance status (Supplementary Table 2).

**Fig. 2:**
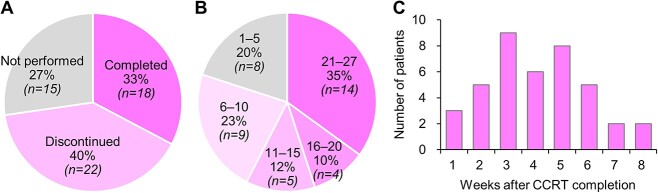
The implementation status of consolidation durvalumab in patients with stage II–III NSCLC treated with CCRT. **A.** Proportions of patients who completed 12 months of durvalumab, discontinued earlier, or did not receive treatment. **B.** Durvalumab cycles administered. **C.** The time from completion of CCRT to initiation of durvalumab.

Consolidation durvalumab was performed in 73% (40/55) of the patients treated with CCRT in 2019 (Fig. 2A). The consolidation treatment was performed for 12 months in 18 patients, whereas the treatment was discontinued earlier in 22 patients (Fig. 2A). Durvalumab was administered for more than 5, 10 and 20 cycles in 32, 23 and 14 patients, respectively. The median number of durvalumab cycles was 12 (Fig. 2B). The time from completion of CCRT to initiation of the first cycle of durvalumab was 4.1 ± 1.8 weeks (Fig. 2C). The reasons for not implementing durvalumab included disease progression, radiation pneumonitis and patient refusal (Supplementary Table 3). The reasons for discontinuing durvalumab earlier than 12 months included disease progression, pneumonitis and poor performance status (Supplementary Table 4). Among 18 patients who completed 12 months of durvalumab treatment, the reasons for the longer durvalumab interval than planned (i.e. every 2 weeks) included pneumonitis and hypothyroidism (Supplementary Table 5).

**Fig. 3:**
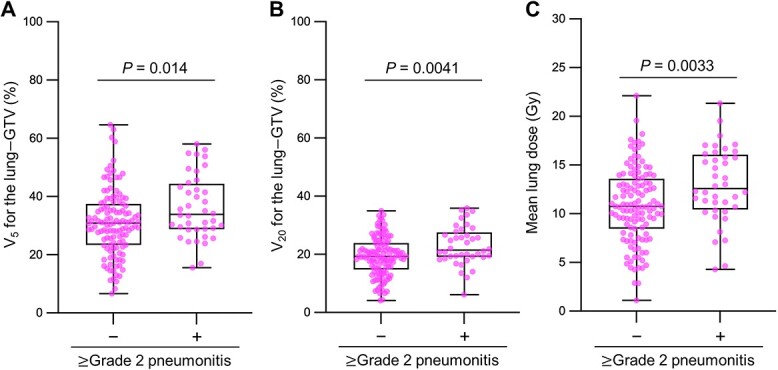
Comparison of dose volume parameters for the lung between patients with stage II–III NSCLC treated with definitive radiotherapy who developed treatment-associated ≥Grade 2 pneumonitis and those who did not. **A.** V_5_ for the lung minus GTV. **B.** V_20_ for the lung minus GTV. **C.** Mean lung dose. *P* values assessed by the Mann–Whitney U test are shown.

**Fig. 4:**
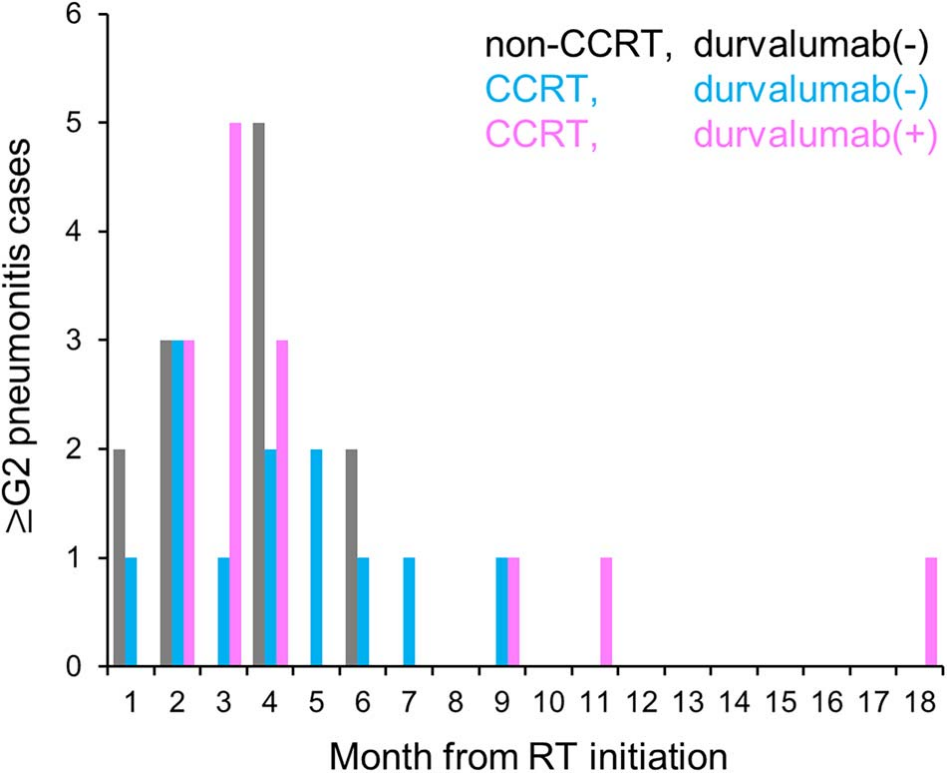
The timing of onset of treatment-associated ≥Grade 2 pneumonitis stratified by treatment modality. CCRT, concurrent chemoradiotherapy; RT, radiotherapy.

Treatment-associated pneumonitis ≥Grade 2 was observed in 23% (39/163) of patients. Pneumonitis ≥Grade 4 was not observed. Regarding the radiation dose, V_5_ for the lung minus GTV, V_20_ for the lung minus GTV, and the mean lung dose were significantly greater in patients who developed ≥Grade 2 pneumonitis than in those who did not (36 ± 11% vs 31 ± 11%, 23 ± 6.7% vs 19 ± 6.7% and 13 ± 3.8 Gy vs 11 ± 3.8 Gy, respectively) (Fig. 3). The effect of concurrent chemotherapy on the onset of pneumonitis was inconclusive (Table 4). The effect of durvalumab on the onset of pneumonitis was not assessed in this survey because it was theoretically impossible to adjust for the bias introduced by the initiation of durvalumab only in patients without pneumonitis ≥Grade 2. Nevertheless, there were no obvious differences in the timing of onset of pneumonitis ≥Grade 2 between the non-CCRT group, the CCRT group treated with durvalumab, and the CCRT group treated without durvalumab; 87% (34/39) of the ≥Grade 2 pneumonitis occurred within 6 months from completion of radiotherapy (Fig. 4).

## DISCUSSION

The results of this study showed that CCRT was the treatment of choice in a significantly greater proportion of stage II–III NSCLC patients in 2019 than in 2017. Meanwhile, the total number of stage II–III NSCLC cases treated with definitive radiotherapy was comparable between the two periods. In 2019, consolidation durvalumab was used in 73% of the CCRT-treated patients, and durvalumab was administered for the planned 12 months and for more than 24 cycles in 45% and 30% of the patients, respectively. To the best of our knowledge, this is the first study analyzing the detailed patterns of care in patients with locally-advanced unresectable NSCLC in relation to the NHI coverage of durvalumab in a real-world prefecture-wide setting with the participation of all the existing radiotherapy facilities. Consolidation durvalumab treatment is covered by the NHI only in a post-CCRT setting. From this perspective, the results of this survey suggest that the NHI coverage of durvalumab had a positive effect on the choice of CCRT as definitive treatment for this disease subset.

Older age was the most frequent reason for not implementing CCRT. The median age of the CCRT group was significantly lower than that of the non-CCRT group in both 2017 and 2019. However, a fraction of elderly patients received CCRT. This suggests that older age can be a confounding factor for other clinical factors in patients who did not receive CCRT, such as the presence of complications or poor performance status, which warrants further detailed investigation. Stage II disease was also a reason for not implementing CCRT. The policy on the use of CCRT in stage II patients might differ among facilities (data not shown) because there are other treatment options besides CCRT (including surgical resection, stereotactic body radiotherapy and external beam radiotherapy alone) that can be considered. The treatment strategy for this disease subset may need further stratification.

**Table 4:** Details of treatment-associated pneumonitis.

Pneumonitis	2017		2019	
	CCRT	non-CCRT	CCRT	non-CCRT
	(*n* = 41)	(*n* = 39)	(*n* = 55)	(*n* = 28)
*Grade 0*	1 (2)	2 (5)	4 (7)	3 (11)
*Grade 1*	32 (78)	25 (64)	32 (58)	25 (89)
*Grade 2*	7 (17)	11 (28)	17 (31)	0 (0)
*Grade 3*	1 (2)	1 (3)	2 (4)	0 (0)

Pneumonitis grades were assessed based on the CTCAE version 4.0. CCRT, concurrent chemoradiotherapy.

This survey revealed the tolerability of consolidation durvalumab post-CCRT in the real-world setting. The results show that one in four patients treated with CCRT was not eligible for consolidation durvalumab. In previous studies, 23–55% of patients with unresectable stage III NSCLC did not meet the criteria of the PACIFIC trial [11, 12, 13, 14]. The treatment was discontinued earlier than the planned 12 months in 55% of patients; this was consistent with the results of the PACIFIC study (51%) [3]. The main reason for discontinuance in this survey was adverse effects (50%); this proportion was slightly higher than that reported in the PACIFIC study (30%). However, the rate of discontinuance due to disease progression was slightly lower in this survey than in the PACIFIC trial (36% vs 61%). Administration of durvalumab every 2 weeks for the planned 12 months should result in at least 24 cycles. In this survey, however, durvalumab was administered for less than 24 cycles in 33% of the patients who completed the 12-month treatment period. A report from Italy showed that 27% of patients completed 1-year durvalumab consolidation, as established by the current national guidelines [15]. This is consistent with the present result of 30% (12/40). Further follow-up is needed to elucidate the effect of a smaller number of durvalumab doses on treatment outcome, as the evidence is lacking on this issue. In the PACIFIC study, initiation of durvalumab within 14 days from the completion of radiotherapy was associated with favorable outcomes. However, some studies reported that the median time to durvalumab initiation was 25–33 days [16, 17]. In this survey, durvalumab was initiated within 14 days from the completion of radiotherapy in only 20% of cases. This may be partially due to the unfamiliarity with the clinical operation of consolidation durvalumab, as the survey was conducted immediately after the start of NHI coverage of this treatment. This could be improved in the near future.

In this survey, the incidence of Grade 2 and Grade 3 pneumonitis was 21% and 2.5%, respectively, whereas ≥Grade 4 pneumonitis was not observed. Among durvalumab-treated patients, the incidence of Grade 2 and Grade 3 pneumonitis was 37% and 2.5%, respectively. In the RTOG0617 study, the incidence of ≥Grade 3 pneumonitis in the standard radiotherapy group receiving 60 Gy was 7% [18]. In the PACIFIC study, on the other hand, the incidence of ≥Grade 3 pneumonitis in durvalumab-treated patients was 3.4% [3]. Oshiro *et al.* reported that the incidence of ≥Grade 2 and ≥ Grade 3 pneumonitis in patients treated with CCRT followed by durvalumab was 34% and 12%, respectively [19]. These data suggest that the incidence of treatment-associated pneumonitis in this survey was comparable to that reported in the literature. With regard to the dose constraints for the lung, the guidelines of the National Comprehensive Cancer Network and those of the European Society for Radiotherapy and Oncology recommend that V_20_ for the lung should be set below 35–40% and 35–37%, respectively, and both guidelines recommend that the mean lung dose should be set below 20 Gy [4, 20]. In this survey, V_20_ for the lung did not exceed 37% in all patients, and the mean lung dose was set below 20 Gy in 98% (2/163) of the patients, suggesting high compliance with the guidelines. The greater incidence of ≥Grade 2 pneumonitis was associated significantly with greater V_5_, V_20_, and the mean dose for the lung, which is consistent with previous studies [21, 22]. Radiation pneumonitis in patients with NSCLC treated with CCRT occurs most often within 6 months from completion of radiotherapy [21]. In this survey, by contrast, three of four patients who developed ≥Grade 2 pneumonitis later than 8 months received consolidation durvalumab. These data indicate that longer follow-up may be needed to determine the incidence of treatment-associated pneumonitis in patients receiving CCRT followed by durvalumab.

This study had several limitations. Firstly, this was a retrospective study. It was difficult to evaluate the impact of other factors that might be influence the treatment choice for CCRT. These factors included radiation technique, renewal of radiation treatment machines, disease state, complication, hospital framework and staffing levels. Secondly, the study was a patterns-of-care survey, and data on long-term treatment outcomes are lacking. Thirdly, the study did not investigate the detailed irradiation strategy, such as elective nodal irradiation or involved field radiotherapy. The irradiation strategy may have affected the lung dose. This issue should be further addressed because the optimal strategy for stage II–III NSCLC remains controversial [23].

In summary, we conducted a region-wide survey to determine the patterns of care for patients with stage II–III NSCLC treated with definitive radiotherapy in the pre- and post-durvalumab era. The results showed that NHI coverage of durvalumab might be a possible reason for choosing CCRT in patients with stage II–III NSCLC. In addition, we demonstrated the tolerability of consolidation durvalumab in the real-world setting. This is the first real-world data on the changing patterns of care for CCRT followed by consolidation durvalumab for NSCLCs, which is important for the clinical management of this patient population in the future.

## Supplementary Material

SupplementaryTable1_rrab116

SupplementaryTable2_rrab116

SupplementaryTable3_rrab116

SupplementaryTable4_rrab116

SupplementaryTable5_rrab116
